# Supplementary material for: Comparison between parameter-efficient techniques and full fine-tuning: A case study on multilingual news article classification
Source: PLoS One. 2024 May 3;19(5):e0301738. doi: 10.1371/journal.pone.0301738 (PMC11068208; doi:10.1371/journal.pone.0301738)
Supplement: S1 Appendix — (PDF) [file pone.0301738.s001.pdf]

S1 Appendix - Sub-task 3 Category Taxonomy

Descriptions of each category are provided in the original task paper [15].

- **Attack on Reputation**
  - Name Calling or Labelling
  - Guilt by Association
  - Casting Doubt
  - Appeal to Hypocrisy
  - Questioning the Reputation
- **Justification**
  - Flag Waving
  - Appeal to Authority
  - Appeal to Popularity
  - Appeal to Values
  - Appeal to Fear, Prejudice
- **Distraction**
  - Strawman
  - Red Herring
  - Whataboutism
- **Simplification**
  - Causal Oversimplification
  - False Dilemma or No Choice
  - Consequential Oversimplification
- **Call**
  - Slogans
  - Conversation Killer

  - Appeal to Time

- **Manipulative Wording**
  - Loaded Language
  - Obfuscation, Intentional Vagueness, Confusion
  - Exaggeration or Minimisation
  - Repetition
