# Supplementary material for: Comparison between parameter-efficient techniques and full fine-tuning: A case study on multilingual news article classification
Source: PLoS One. 2024 May 3;19(5):e0301738. doi: 10.1371/journal.pone.0301738 (PMC11068208; doi:10.1371/journal.pone.0301738)
Supplement: S2 Appendix — (PDF) [file pone.0301738.s002.pdf]

S2 Appendix - ablation study for PEFT techniques

In this section, we perform the ablation study to find how each of three PEFT techniques performs in a multilingual, cross-lingual and monolingual scenarios on sub-task 1. As can be seen, while LoRA and Adapter often demonstrate comparable behaviour in all training scenarios, the BitFit method achieves significantly lower results. Importantly, while the results in the multilingual setting are comparable, the difference between BitFit and two other PEFTs is particularly noticeable for the two cross-lingual settings ('English + Translations' and 'English Only') scenarios.

Table S2. Sub-task 1: Genre Detection - Mean ± 1 STD $F1_{macro}$ scores for three PEFT techniques in 3 training scenarios.

Language	Multilingual Joint			English + Translations			English Only		
	BitFit	LoRA	Adapter	BitFit	LoRA	Adapter	BitFit	LoRA	Adapter
EN	51.6±0.8	49.4±0.4	**52.8±0.2**	50.2±2.1	52.4±0.6	***53.5±0.9**	40.3±1.7	39.1±1.2	**42.2±0.6**
FR	65.5±0.9	67.4±2.3	**67.5±0.9**	68.0±1.2	***69.2±1.5**	68.4±0.7	65.4±1.5	66.3±0.5	**66.7±3.7**
DE	64.2±0.7	64.8±1.2	***67.2±0.8**	64.1±1.5	63.9±3.0	**65.8±1.2**	60.3±5.2	**64.2±2.8**	63.6±0.7
IT	51.0±1.6	***53.4±1.8**	52.0±3.1	49.5±1.3	52.0±1.5	**52.9±0.8**	42.1±2.4	**47.3±1.8**	44.2±1.1
PL	63.6±1.7	***66.8±0.4**	65.2±1.5	58.1±0.5	**64.0±0.7**	61.8±2.4	58.2±0.7	**60.6±1.4**	59.6±3.0
RU	53.1±0.9	***55.7±1.7**	52.8±0.9	51.4±1.2	54.2±0.8	**54.9±2.5**	43.7±1.8	**49.4±2.4**	48.7±0.6
Average	58.13±1.6	***59.6±1.9**	***59.6±3.1**	56.9±2.6	59.3±5.2	***59.6±2.7**	51.3±3.8	54.5±6.1	**54.2±2.9**
ES	42.4±1.6	41.8±0.5	**44.2±0.7**	44.0±1.8	46.0±1.3	***46.2±5**	40.3±0.7	**41.7±0.8**	40.9±1.1
EL	40.1±1.3	**41.4±2.7**	40.9±1.7	39.1±1.1	***42.9±1.7**	42.2±3.6	**36.7±1.3**	38.6±1.7	37.5±0.8
KA	78.5±2.2	***80.8±5.0**	79.2±1.8	77.7±2.4	**79.6±4.1**	77.5±2.2	71.3±2.9	72.9±1.5	**74.8±2.4**
Average	53.6±3.1	54.7±2.4	**54.8±1.8**	53.2±2.4	***56.2±3.3**	55.3±1.9	49.2±2.7	**51.1±4.0**	**51.1±3.3**
All	56.3±4.1	57.9±6.3	**58.0±2.0**	55.2±3.7	***58.2±3.8**	58.1±4.1	50.6±4.4	**53.3±2.6**	53.1±3.6

Best scores by language are marked with an asterisk (∗). Best scores by training method for each training scenario are in **bold**.
