# Supplementary material for: Comparison between parameter-efficient techniques and full fine-tuning: A case study on multilingual news article classification
Source: PLoS One. 2024 May 3;19(5):e0301738. doi: 10.1371/journal.pone.0301738 (PMC11068208; doi:10.1371/journal.pone.0301738)
Supplement: S3 Appendix — (PDF) [file pone.0301738.s003.pdf]

S3 Appendix - Model size selection for XLM-RoBERTa

The results of our comparison between different sizes of RoBERTa model are provided in S3.1, S3.2, S3.3 Tables. As can be seen, for all training techniques, the average results are consistently significantly higher for the large size of the model for all three sub-tasks.

Table S3.1. Comparison of the Base and Large sizes of the model in the 'Multilingual Joint' scenario for the FFT method

Language	Sub-task 1		Sub-task 2		Sub-task 3	
	XLMR-Base	XLMR-Large	XLMR-Base	XLMR-Large	XLMR-Base	XLMR-Large
EN	35.2±1.8	52.7±0.5	50.9±1.1	55.8±0.2	26.4±0.8	34.9±1.7
FR	69.7±0.6	69.7±1.2	44.4±2.5	53.3±3.3	34.2±1.4	45.9±1.2
DE	67.2±2.2	66.3±0.5	60.7±1.0	63.1±1.9	41.4±1.3	52.1±1.9
IT	44.5±2.1	52.2±1.4	53.4±2.5	59.9±1.9	46.2±0.7	55.1±2.5
PL	68.7±3.0	69.2±1.1	60.1±3.5	65.2±0.8	27.6±1.2	40.4±3.2
RU	55.2±1.4	57.4±0.6	42.2±3.0	45.3±3.0	29.6±2.1	40.9±1.4
ES	40.8±2.4	47.1±1.4	51.9±1.7	52.7±2.1	28.3±1.4	37.7±2.3
EL	43.3±3.9	40.8±2.4	48.9±1.1	54.9±1.7	21.1±2.7	26.7±0.9
KA	77.5±1.8	83.3±2.1	49.7±2.6	60.1±4.2	33.6±2.7	42.6±1.0
all	55.2±6.1	59.9±3.1	51.4±6.2	56.7±6.1	32.1±7.8	41.8±8.6

Table S3.2. Comparison of the Base and Large sizes of the model in the 'Multilingual Joint' scenario for LoRA method

Language	Sub-task 1		Sub-task 2		Sub-task 3	
	XLMR-Base	XLMR-Large	XLMR-Base	XLMR-Large	XLMR-Base	XLMR-Large
EN	45.2±2.6	49.4±0.4	44.9±3.4	52.2±1.7	31.4±0.8	37.7±0.9
FR	62.1±0.9	67.4±2.3	43.8±0.5	47.3±1.5	42.6±1.1	48.6±0.8
DE	60.2±1.6	64.8±1.2	50.8±1.4	62.3±2.2	48.2±1.3	52.3±0.9
IT	49.4±1.5	53.4±1.8	54.1±1.2	56.8±1.6	50.4±1.9	58.7±0.5
PL	60.2±2.0	66.8±0.4	56.7±1.8	61.0±0.8	40.3±0.7	42.1±0.6
RU	52.1±1.5	55.7±1.7	35.7±0.7	43.6±0.6	33.4±1.4	42.3±0.3
ES	33.5±1.7	41.8±0.5	46.5±2.1	51.7±3.0	44.2±1.2	39.0±1.6
EL	37.8±0.7	41.4±2.7	44.9±2.1	51.4±1.2	23.6±2.2	25.5±0.6
KA	75.1±3.3	80.8±5.0	48.1±2.5	53.9±4.8	37.9±2.4	40.4±3.1
all	52.8±4.5	57.9±6.3	47.5±2.8	53.4±6.0	39.7±4.7	42.9±9.5

Table S3.3. Comparison of the Base and Large sizes of the model in the 'Multilingual Joint' scenario for the adapter method

Language	Sub-task 1		Sub-task 2		Sub-task 3	
	XLMR-Base	XLMR-Large	XLMR-Base	XLMR-Large	XLMR-Base	XLMR-Large
EN	44.5±1.8	52.8±0.2	53.4±2.5	55.7±2.0	30.6±3.1	37.5±2.9
FR	58.8±2.4	67.5±0.9	46.4±1.2	50.8±3.6	37.2±4.1	45.7±1.9
DE	61.7±2.1	67.2±0.8	55.0±1.1	64.2±1.0	38.3±1.9	53.0±0.7
IT	43.9±3.5	52.0±3.1	56.6±1.7	58.2±1.0	50.4±2.2	58.1±1.6
PL	59.2±1.5	65.2±1.5	61.1±3.0	64.1±1.7	35.2±2.7	41.9±2.7
RU	42.2±1.8	52.8±0.9	37.5±1.2	41.7±2.0	28.9±3.1	39.2±2.6
ES	39.8±2.7	44.2±0.7	40.6±3.3	49.1±2.0	28.3±1.5	36.7±1.3
EL	35.7±2.1	40.9±1.7	44.2±1.4	54.1±2.9	21.6±1.7	25.4±1.5
KA	71.5±2.2	79.2±1.8	47.3±2.4	55.3±1.6	37.1±1.6	42.2±2.4
all	51.5±3.1	58.0±2.0	49.4±5.2	54.8±7.1	34.8±6.6	42.2±9.5
