# Supplementary material for: Comparison between parameter-efficient techniques and full fine-tuning: A case study on multilingual news article classification
Source: PLoS One. 2024 May 3;19(5):e0301738. doi: 10.1371/journal.pone.0301738 (PMC11068208; doi:10.1371/journal.pone.0301738)
Supplement: S4 Appendix — (PDF) [file pone.0301738.s004.pdf]

S4 Appendix - Amount of resources per-language

S3 Table shows the amount of resources we considered when identifying low-resource languages for our task. For the amount of pre-training data, we summarised the statistics from [43]. We also considered the existence of the data for fine-tuning in a certain language or whether training data is available in a language from the same group (column named ”Language family”). Based on our criteria, Georgian language is a clear outlier with substantially less pre-training data and no training data in that language.

Table S4. Comparison of the amount of resources per each language in sub-tasks 1, 2, and 3

Language	Pre-training data (number of tokens)	Training data (number of examples)			Language family
		sub-task1	sub-task2	sub-task3	
EN	55,608	433	433	3,610	West-Germanic
FR	9,780	158	158	1,693	Romance
DE	10,297	132	132	1,251	West-Germanic
IT	4,983	226	227	1,742	Romance
PL	6,490	144	145	1,228	Slavic
RU	23,408	142	143	1,232	Slavic
ES	9,374	0	0	0	Romance
EL	4,285	0	0	0	Hellenic
KA	469	0	0	0	Kartvelian
