# Supplementary material for: Comparison between parameter-efficient techniques and full fine-tuning: A case study on multilingual news article classification
Source: PLoS One. 2024 May 3;19(5):e0301738. doi: 10.1371/journal.pone.0301738 (PMC11068208; doi:10.1371/journal.pone.0301738)
Supplement: S5 Appendix — (PDF) [file pone.0301738.s005.pdf]

S5 Appendix - Comparison of Houlsby and Pfeiffer adapters across three sub-tasks

S4 Table shows the comparison of Housby and Pfeiffer configuration of the adapter for each sub-task in our experiments in a 'Multilingual Joint' training scenario for the FFT method. As can be seen, the differences are not consistent across all the languages, but on average, each sub-task benefits from the Pfeiffer adapter setting.

Table S5. Comparison of the Houlsby and Pfeiffer adapters for XLM-RoBERTa Large in the 'Multilingual Joint' scenario

Language	Sub-task 1		Sub-task 2		Sub-task 3	
	Housby	Pfeiffer	Houlsby	Pfeiffer	Houlsby	Pfeiffer
EN	43.2±5.2	52.8±0.2	54.3±2.3	55.7±2.0	36.2±2.3	37.5±2.9
FR	66.7±1.3	67.5±0.9	47.4±4.8	50.8±3.6	47.7±1.0	45.7±1.9
DE	68.1±0.5	67.2±0.8	63.5±2.1	64.2±1.0	52.6±0.4	53.0±0.7
IT	50.7±2.4	52.0±3.1	59.0±1.1	58.2±1.0	55.1±1.3	58.1±1.6
PL	64.0±3.1	65.2±1.5	62.6±0.8	64.1±1.7	42.5±0.7	41.9±2.7
RU	59.7±1.9	52.8±0.9	40.0±2.2	41.7±2.0	40.5±0.6	39.2±2.6
ES	47.2±1.7	44.2±0.7	50.4±2.1	49.1±2.0	38.5±2.2	36.7±1.3
EL	44.5±3.6	40.9±1.7	52.0±2.7	54.1±2.9	27.6±0.8	25.4±1.5
KA	76.1±0.4	79.2±1.8	56.5±3.1	55.3±1.6	43.0±2.7	42.2±2.4
all	57.9±5.5	58.0±2.0	54.0±8.0	54.8±7.1	42.1±8.6	42.2±9.5
